# Increase of Vascular Endothelial Growth Factor and Decrease of MCP-1 and Some Updated Epidemiology Aspects of Cystic Echinococcosis Human Cases in Calabria Region

**DOI:** 10.1155/2018/4283672

**Published:** 2018-01-14

**Authors:** Giovanni Matera, Maria Teresa Loria, Cinzia Peronace, Tatiana Catanzariti, Pio Settembre, Aida Giancotti, Angelo G. Lamberti, Giorgio S. Barreca, Luisa Galati, Gessica Dodaro, Maria Mazzitelli, Alessio Strazzulla, Carlo Torti, Angela Quirino, Maria Carla Liberto, Alfredo Focà

**Affiliations:** ^1^Institute of Microbiology, Department of Health Sciences, “Magna Græcia” University of Catanzaro, Viale Europa, 88100 Catanzaro, Italy; ^2^Infection Disease Unit, Department of Medical and Surgical Sciences, “Magna Græcia” University of Catanzaro, Viale Europa, 88100 Catanzaro, Italy

## Abstract

We aim to investigate some of the pathogenetic mediators of the human echinococcosis and to obtain updated epidemiological findings on cases of echinococcosis in Calabria, Southern Italy. Echinococcosis diagnosis was based on imaging, serological investigations, and molecular assay. Indeed, real-time PCR indicated the presence of G2/G3 genotypes of *Echinococcus granulosus* complex. Regarding pathogenesis, a relevant novel tool of immune depression should be deemed the reduced level of serum MCP-1. Also, we found a previously unreported VEGF, possibly associated with neovascularization requested by the parasite cyst metabolism. Cytokine profiles suggest a bias of the immunity toward Th2 and Treg responses. Nitric oxide levels exhibited a significant decrease one week after therapy versus basal level measured before surgery and/or chemotherapy. An increase of serum total IgE class and IgG4 subclass was found in *Echinococcus*-positive patients versus controls. Our data demonstrated an endemic spreading, at least in the province of Catanzaro and neighboring Calabria territories, for such parasitosis with the novel issue of the number of female overcoming male cases. In conclusion, the novel findings of this study were the increased VEGF and the reduced serum MCP-1 in the studied cases, as well as the number of *Echinococcus*-infected females overcoming the infected males.

## 1. Introduction

Cystic echinococcosis (CE) is an important cestode infection which has been reported as one of the most prevalent zoonoses worldwide [1, 2]. Cystic echinococcosis (CE) is a chronic infectious disease caused by the larval stage of cestode *Echinococcus granulosus* and reflecting a peculiar host/parasite interaction in humans. The taxonomy of species/genotypes causing cystic echinococcosis includes the *E. granulosus* sensu lato (s.l.) complex groups, *E. granulosus* sensu stricto (s.s.) (G1/G2/G3), *E. equines* (G4), *E. ortleppi* (G5), *E. canadensis* (G6/G7/G8/G10), and *E. felidis* (“lion strain”) [3].

CE is a potentially severe disease and may involve vital organs such as the liver, lung, and brain. It is primarily a disease of herbivorous animals, and man is infected accidentally, through ingestion of food contaminated by fecal material from *Echinococcus* definitive hosts (e.g., dogs, wolves, and foxes) [4]. Clinical features of the disease may often change and depend on the organs involved, the size of cysts and their sites within the affected organ, complications caused by rupture of cysts, and subsequent often fatal immunologic reactions [5, 6].

From the literature, it is well known that pathogenesis mechanisms associated with *Echinococcus* infections may be exploited to assess the relationships between contrasting forces played by cellular and humoral immunostimulant and immunosuppressive mediators. It has been reported that inhibitory activity of Th2 and Treg immune responses may play a pivotal role in the evasion of host defenses, leading to persistent worm infections. The roles of cytokines and other mediators of host immunity seem to be quite complex in echinococcosis and may differ with species of helminth, with size, viability and location of cyst within the host, the products of its metabolism, and species of the host [5, 7, 8]. Moreover, conflicting cytokine findings have been reported by different investigators [9]. Although nitric oxide has been associated with parasiticidal effects on both clinical and experimental settings, very few observations have been published on nitric oxide during *Echinococcus* infections in humans.

Epidemiology of *Echinococcus* has been thoroughly investigated in Europe and some regions of Italy. On the contrary, very scanty publications and data are dealing with *Echinococcus* epidemiology in Calabria, the southernmost region of continental Italy, where sheep, goat, and cattle breeding is even presently widespread, as discussed by Tamarozzi et al. [10].

Therefore, the aims of the present study are the evaluation of some epidemiological aspects of echinococcosis in Calabria region, as well as of the profile of cytokines and other primary humoral mediators during *Echinococcus* infections.

## 2. Materials and Methods

### 2.1. Subjects, Parasite Materials, and Serum Samples

During the last ten years, a total of 53 patients were diagnosed as infected by *Echinococcus* spp. by ultrasound (US) examination and serological methods, in the Catanzaro University Hospital. Among such *Echinococcus*-positive patients, serum samples and hydatid cyst liquid were available only for 19 subjects, after surgery or PAIR (puncture, aspiration, injection of a protoscolicidal agent, and reaspiration) technique. Moreover, 10 control *Echinococcus*—not infected—patients with nonparasitic liver cysts (Echin Neg), as well as 12 healthy volunteers (CON), were included as controls in this study. Subjects of both control groups were checked by US and serological tests, and those *Echinococcus*-positive for one of such diagnostic methods were excluded from these control groups. Other exclusion criteria for all enrolled subjects were the presence of neoplastic diseases, severe metabolic alterations, and major liver and kidney impairments.

The study protocol was approved by the Ethical committee of the University of Catanzaro, and all subjects gave their informed written consent.

### 2.2. Microscopic Examination

Microscopy of surgically obtained cyst liquid was carried out after centrifugation of some aliquots of specimens. Microscopy slides were prepared with saline or stained with gram stain and observed by light microscopy. Other preparations were observed by fluorescent microscopy [11] with or without fluorescent dye (acridine orange).

### 2.3. Molecular Biology

#### 2.3.1. DNA Extraction

Cystic liquid from hydatid cysts was collected and stored at −20°C until DNA extraction. DNA was extracted using a QIAamp DNA Mini Kit (Qiagen, Hilden, Germany) following a modified procedure; an aliquot of 500 *μ*l of specimens was centrifuged (1500*g* for 5 min), and the pellet was resuspended in 180 *μ*l of QIAamp ATL buffer with 400 *μ*g proteinase K and DNA extracted following the manufacturer recommendations and eluted from the kit columns to give a final volume of 100 *μ*l [12].

EasyMag (bioMérieux, Italy) was used for automated extraction from 500 *μ*l blood sample. DNA sample was eluted to a final volume of 55 *μ*l.

#### 2.3.2. Real-Time PCR Assay

The LightCycler system (Roche Diagnostics, Italy) with LightCycler FastStart DNA Master SYBR Green I was used for amplification and real-time detection. For genus-specific real-time PCR assay, the forward 12S F GTTAAGCTAAGTCTATGTGCTGC and reverse primers12S R CTCTCTTCACATCAACAAACTCATTTAA [13] were used to amplify a 126 bp portion of the 12S mtDNA gene. PCR mixture contained 2 *μ*l of extracted DNA, 0.25 *μ*l of each primer, 1.2 *μ*l of MgCl2, 2 *μ*l of SYBR Green, and 14.3 *μ*l of H_2_O for PCR supplied by the kit. The amplification PCR conditions were activation of the polymerase enzyme (95°C for 3 min), and 40 cycles of amplification were performed, each one including 94°C for 30 s, 62°C for 30 s, and 72°C for 30 s, followed by a final extension at 72°C for 7 min. Fluorescence signals were measured once in each cycle at the end of the extension step. The melting experiment was performed from 45°C to 99°C at 0.2°C/s with continuous fluorescence monitoring. After amplification, melting curve analysis was carried out by evaluating a Tm (melting temperature) of 76.4 ± 0.15°C for genotype G1 and 77.0 ± 0.13 for genotype G2/G3.

Each PCR run included a negative extraction control (sterile water) and a negative PCR control containing 5 *μ*l DEPC-treated H_2_O instead of DNA extract, to verify any possible presence of contaminating DNA in the test. Samples and controls were run in duplicate.

### 2.4. Determination of Nitric Oxide (NO)

Nitrite, the primary, stable, and nonvolatile product of NO, was quantified as an indirect correlate of NO production. The patient sera were analyzed for nitrite contents by the method described by Miranda and colleagues [14]. Briefly, the Griess reagent (1% sulfanilamide and 0.3% N-(1-naphthyl)ethylenediamine dihydrochloride in 5% H_3_PO) was added to aliquots of patient serum with a reducing agent (vanadium HCl) and incubated for 30 min at 37°C. Then, optical density (OD) was determined at 540 nm using a spectrophotometer. Sodium nitrite (NaNO_2_), diluted in serum of a healthy volunteer, was used to generate a standard curve [14].

### 2.5. Serological Methods

Peripheral blood samples were obtained to carry over serological tests. One of these assays was based on searching specific anti-*Echinococcus* Ab of IgG class with immunoenzymatic method (ELISA-NovaTec). The other technique used was the IDA test (Hydatidose Fumouze, Diagnostics) which is based on indirect haemagglutination. Sensitized red blood cells are composed of sheep red blood cells coated with *Echinococcus granulosus* antigen. Serum antibodies against *Echinococcus granulosus* are revealed by agglutination of the sensitized red blood cells: a reddish-brown film can be observed in the positive sample well. In the absence of specific antibodies (negative test), these red blood cells are expected to form a ring in well bottom.

Total IgG and IgG4 subclasses, as well as C-reactive protein (CRP), were analyzed by nephelometer (BNTM II system immunonephelometry) with high levels of sensibility and specificity. Total IgE antibodies were assayed by IgE immunoenzymatic technique (Radim SpA, Pomezia, Italy).

### 2.6. Measurement of Serum Cytokines with Biochip Array

To carry out quantitation of cytokines, a technology based on Biochip Array was used. It refers to a “sandwich” immunoassay with a chemiluminescent detection system. Thus, it is possible to measure all analytes on a single biochip simultaneously. Several Th1, Th2, and Treg cytokines, chemokines, and growth factors were evaluated by “Evidence Investigator” semiautomatic instrumentation by the panel “cytokine kit” (Randox Laboratories Ltd., Crumlin, UK).

The analyzer provided a measure for the chemiluminescence through a CCD camera (charge-coupled device) and converted light signals into data through a dedicated software.

### 2.7. Statistical Analysis

Quantitative data obtained were expressed as means ± SEM. Analysis of variance (ANOVA) and Fisher's Protected Least Significant Difference (Fisher's PLSD) post hoc test was used to evaluate significant differences among groups. A value of *p* < 0.05 was considered significant.

## 3. Results and Discussion

The total number of patient parasite-positive whose samples arrived at our laboratory over the past 10 years was equal to 53.

For the present study, there were available serum samples and cystic liquid (frozen at −20°C) obtained from 19 patients with one or more *Echinococcus* cysts, confirmed by diagnostic imaging techniques (ultrasound, CT SCAN, and MRI) and/or serological investigations. According to WHO-IWGE classification [4], all our patients exhibited cysts belonging to transitional stage (CE3a/CE3b).

The demographic data and laboratory findings of 19 patients from the province of Catanzaro and some neighboring areas studied during the period of 2012–2016 are shown in Table 1. Interestingly, the ratio between infected females and males was 10 : 9.

**Figure 1:**
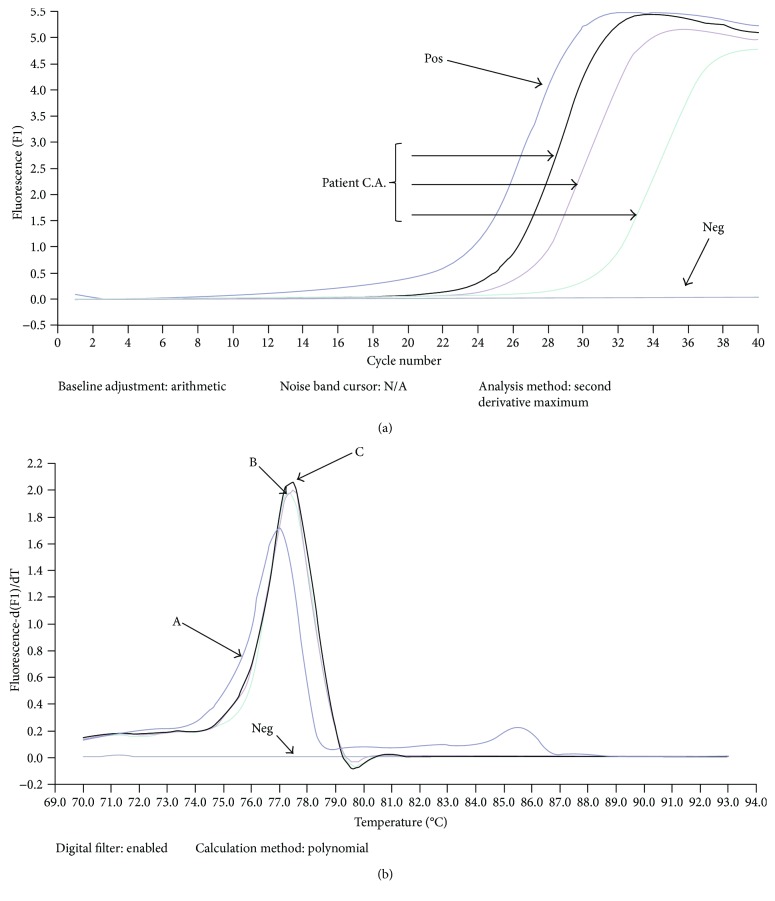
(a) PCR real-time curves obtained from cystic liquid of our patient C.A. following the procedure of Capuano et al. [12] reported in “Material and Methods”; (b) melting curves of hydatid cyst of standard *E. granulosus* genotypes G1 with Tm 76.55 (A), genotypes G2/G3 with Tm 77.03 (B), and of hydatid cyst of patient C.A. with Tm 77.11 (C) from this study. Neg is a negative control.

From cystic liquid samples of our patients, molecular assays were carried out to further characterize the pathogen and to evaluate its genotypes. PCR real-time curves, following the procedure of Maurelli et al. [13], obtained from cystic liquid of our patient C.A., are shown in Figure 1. All the positive samples analyzed exhibited melting curves overlapping those shown in Figure 1 and belonged to genotype G2/G3.

**Figure 2:**
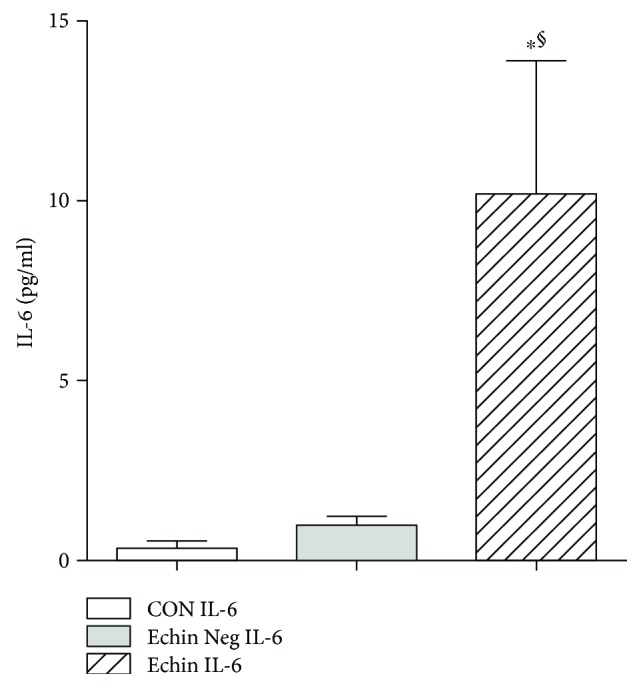
Serum levels of IL-6 in the group of infected patients (Echin IL-6) are significantly increased (*p* < 0.05) in comparison to the IL-6 concentration evaluated in the group of *Echinococcus*-negative patients (Echin Neg IL-6) and the group of control subjects (CON IL-6). ^∗^*p* < 0.05 versus CON; ^§^*p* < 0.05 versus Echin Neg, after ANOVA and Fisher PLSD test.

**Figure 3:**
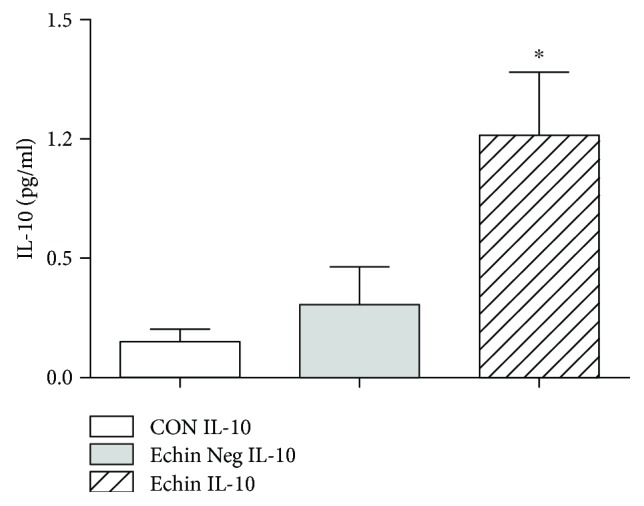
Serum levels of IL-10 in the group of infected patients (Echin IL-10) are significantly increased (*p* < 0.05) in comparison to the IL-10 concentration evaluated in the group of *Echinococcus*-negative patients (Echin Neg IL-10) and in the group of control subjects (CON IL-10). ^∗^*p* < 0.05 versus CON, after ANOVA and Fisher PLSD test.

**Figure 4:**
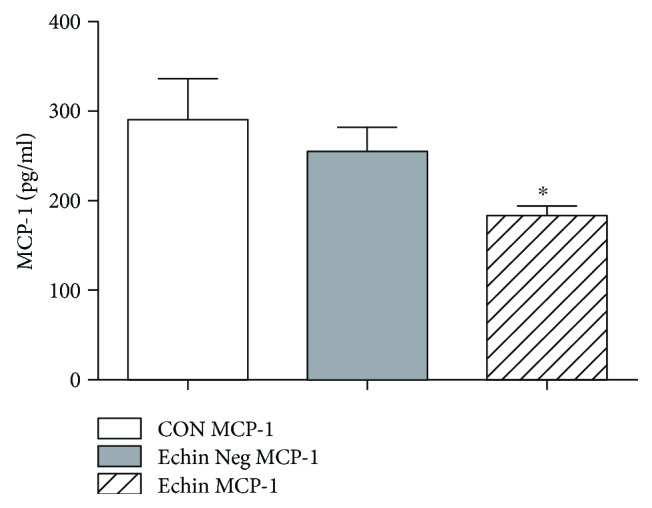
Serum levels in the group of infected patients (Echin MCP-1) are significantly decreased (*p* < 0.05) versus the correspondent parameters in the group of *Echinococcus*-negative patients (Echin Neg MCP-1) and in the group of control subjects (CON MCP-1). ^∗^*p* < 0.05 versus CON, after ANOVA and Fisher PLSD test.

**Figure 5:**
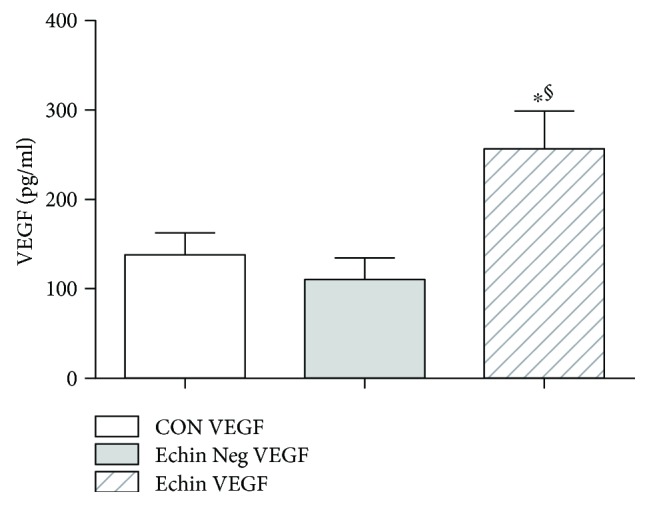
Serum levels of VEGF in the group of infected patients (Echin VEGF) are significantly increased (*p* < 0.05) versus the correspondent parameters in the group of *Echinococcus*-negative patients (Echin Neg VEGF) and in the group of control subjects (CON VEGF). Values are expressed as mean ± SEM. ^∗^*p* < 0.05 versus CON; ^§^*p* < 0.05 versus Echin Neg, after ANOVA and Fisher PLSD test.

With regard to the evaluation of the cytokine profile in the course of echinococcosis, we observed a significant increase of interleukin 6 (IL-6) (*p* < 0.05; Figure 2) and interleukin 10 (IL-10) (*p* < 0.05; Figure 3), which may suggest an increase in Th2 and Treg responses. A significant decrease of MCP-1 (*p* < 0.05; Figure 4) and a significant increase of vascular endothelial growth factor (VEGF) were also found (*p* < 0.05; Figure 5).

**Figure 6:**
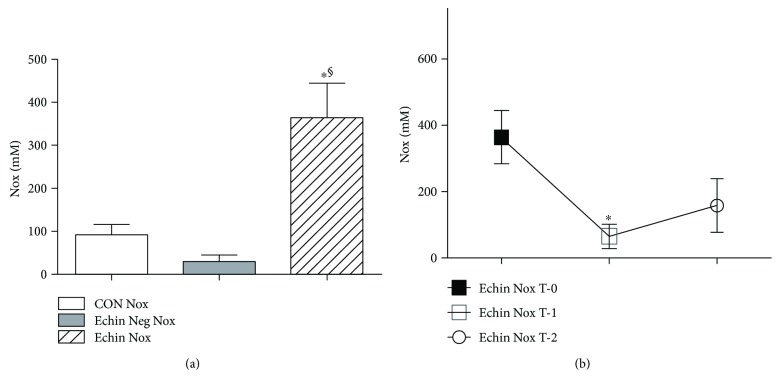
(a) Serum levels of Nox showed a significant increase (*p* < 0.05) compared to corresponding serum concentrations found in healthy controls and *Echinococcus*-negative patients. Values are expressed as mean ± SEM. ^∗^*p* < 0.05 versus CON; ^§^*p* < 0.05 versus Echin Neg, after ANOVA and Fisher PLSD test. (b) Significant decline in the concentrations of nitric oxide metabolites in parasite-infected patients one week after surgery (time T-1) (*p* < 0.05) compared with the levels found in patients before any therapy (time T-0); however, 1-2 months after therapy (time T-2), such nitric oxide metabolites exhibited a light increase.

In patients with echinococcosis, before any surgical and/or medical therapy, evaluation of the metabolites (NO_3_ and NO_2_) of nitric oxide exhibited a significant increase versus values found in healthy volunteers and *Echinococcus*-negative patients (*p* < 0.05; Figure 6(a)).The nitric oxide showed a significant decline in the concentrations of these metabolites in parasite-infected patients one week after surgery (time T-1) (*p* < 0.05) compared with the levels found in patients before any therapy (time T-0). However, 1-2 months after therapy (time T-2), such nitric oxide metabolites exhibited a light increase (Figure 6(b)).

**Figure 7:**
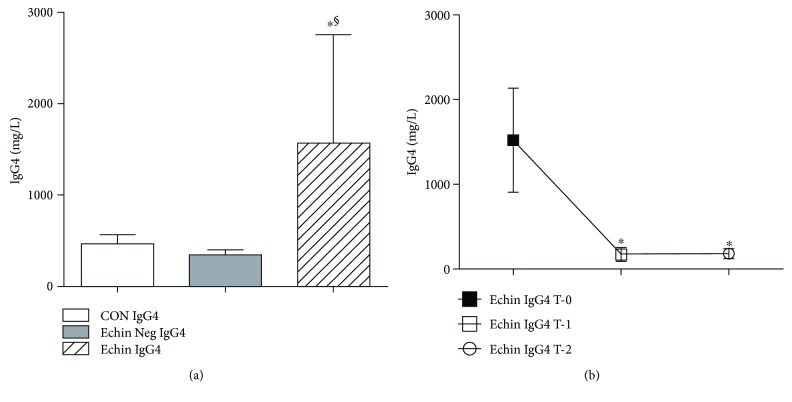
(a) Serum levels of IgG4 in the group of infected patients (Echin IgG4) are significantly increased (*p* < 0.05) versus the same parameters in the group of Echin-negative patients (Echin Neg IgG4) and in the group of control subjects (CON IgG4). Values are expressed as mean ± SEM. ^∗^*p* < 0.05 versus CON; ^§^*p* < 0.05 versus Echin Neg, after ANOVA and Fisher PLSD test. (b) IgG4 levels showed a significant decrease in parasite-infected patients one week after surgery (time T-1) (*p* < 0.05) compared with the levels found in patients before any therapy (time T-0); such reduction was observed even 1-2 months after therapy (time T-2). ^∗^*p* < 0.05 versus Echin IgG4 T-0, after ANOVA and Fisher PLSD test.

For some of the patients suffering from echinococcosis and discussed further below, the dosage of total IgG and some IgG subclasses was performed. As regards the levels of total IgG, no difference was observed between the group of patients with echinococcosis, parasite-negative patients, and healthy volunteers. On the contrary, the value of IgG4 in worm-infected patients was higher about three times in comparison to healthy volunteers and *Echinococcus*-negative patients (*p* < 0.05; Figure 7(a)). IgG4 levels showed a significant decrease in parasite-infected patients one week after surgery (time T-1) (*p* < 0.05) compared with the levels found in patients before any therapy (time T-0); such reduction was observed even 1-2 months after therapy (time T-2) (Figure 7(b)).

**Figure 8:**
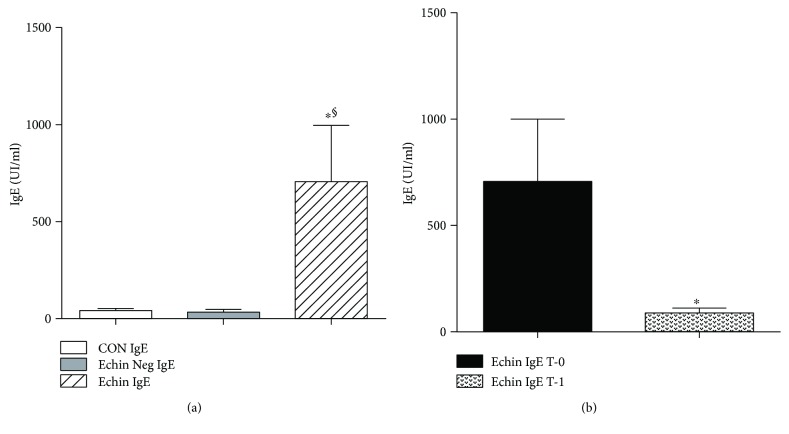
(a) Serum levels of IgE in the group of infected patients showed a significant increase (*p* < 0.05) compared to corresponding serum concentrations found in healthy controls and *Echinococcus*-negative patients. Values are expressed as mean ± SEM ^∗^*p* < 0.05 versus CON; ^§^*p* < 0.05 versus Echin Neg, after ANOVA and Fisher PLSD test. (b) A significant decline in the concentrations of levels of IgE in parasite-infected patients one week after surgery (time T-1) (*p* < 0.05) compared with the levels found in patients before any therapy (time T-0). ^∗^*p* < 0.05 versus Echin IgE T-0, after ANOVA and Fisher PLSD test.

Moreover, total IgE levels were significantly (*p* < 0.05) higher in the *Echinococcus*-infected patients versus *Echinococcus*-negative, as well as the control group. Other investigators found that total IgE levels were significantly different between *Echinococcus* cyst patients and healthy controls. In our study, we made a comparison between *Echinococcus*-positive patients and nonparasitic cyst subject, as well as healthy controls, and only the first group revealed a significantly higher level of total IgE (Figure 8(a)). However, such high level of total IgE was found significantly reduced following surgical or medical therapy (Figure 8(b)).

A thorough analysis of the scientific literature revealed a lack of epidemiological studies concerning the incidence and prevalence of the infection by *Echinococcus* in Calabria region. The present study might suggest an endemic spreading in the province of Catanzaro and neighboring territories for such parasitic disease. Also, it would seem that the greater density of cases of echinococcosis occurs in the area of Soverato (close to the city of Catanzaro) and Marchesate (particularly around the towns of Cirò and Crotone).

The average age of parasite-positive patients was found to be 55.3, while the ratio of males to females was 1 : 9. The relationship between the sexes is a new finding. Recent publications on the topic by Italian authors had shown that in regions of high endemicity (Sardinia, etc.) the relationship between the sexes was constantly in favor of men. This finding was explained due to the higher frequency of shepherds among male patients [15]. On the contrary, in our study, the number of male and female patients are very close, and the majority of female patients were housewives, and this observation might drive the attention on different risk factors (e.g., contact with pet dog; processing of contaminated fruits and other fresh produces in both home and work settings).

Regarding the species of *Echinococcus*, only *E. granulosus* has been identified. Furthermore, the molecular characterization of some isolates showed only the genotype G2/G3, belonging to the taxonomic group reported as *E. granulosus* sensu strict [3, 16].

However, the recent identification of numerous foxes, also in the urban territory of the province of Catanzaro, does not allow to exclude the possible involvement of different *Echinococcus* species such as *E. multilocularis.* This species has been identified in territories of northern Italy, while seems to be absent in some regions of central Italy [17].

Our study findings show that 26% of *Echinococcus*-infected patients were negative for *Echinococcus*-specific antibodies by the IDA test, which is based only on agglutinating antibodies (regardless the class/subclass of such agglutinating antibodies) that represent the screening test for the serology of the echinococcosis. It has been reported that up to 30–40% of cystic echinococcosis cases are antibody-negative [5].

IgG antibodies are distinguished into four subclasses: IgG1, IgG2, IgG3, and IgG4. The differences between IgG subclasses are reflected in several important biological functions, such as the antigenic recognition, the complement activation, and the type of bond with the receptors on the cell surface [5]. Many studies have demonstrated that changes in concentrations of IgG subclasses in serum, compared to the normal values, are connected to different kinds of diseases: variations in the concentration of the IgG subclasses were observed in patients with parasitic diseases including echinococcosis [18]. It has also been found the presence of a concentration of IgG4 substantially higher in parasite-positive patients in comparison with controls. The absence of IgG antibodies in the presence of cysts was already published [5, 18]. Also, the increase of the subclass IgG4 in the course of echinococcosis was already reported in the literature [5, 18]. However, this subclass of IgG was often associated with the immune depression involving T and B lymphocytes and other cell types of immune defense. Recently, using an in vitro human model, it was found that IgG4 production is specifically confined to IL-10 BR1 cells [19]. It is tempting to speculate that *Echinococcus* specifically stimulates the release of immunoglobulins IgG4 which is associated with a negative modulation of immunity. This could certainly be a parasite mechanism to circumvent host defenses.

Previously, our group studied the immunodepression associated with infections by helminths in a clinical setting of atopic patients and demonstrated the improvement of the allergic pathology during worm infection, while the Ascaris eradication led to re-emergence of allergic symptoms [20].

Other investigators found that total IgE levels were significantly different between *Echinococcus* cyst patients and healthy controls [21]. In our study, we made a comparison between *Echinococcus*-positive patients and nonparasitic cyst subject, as well as healthy controls, and only the first group revealed a significantly higher level of total IgE. However, such high level of total IgE was found significantly reduced following surgical or medical therapy. Therefore, total IgE can be used as both diagnostic and prognostic markers in the management of *Echinococcus* patients.

Interleukin-6 (IL-6) is a pleiotropic cytokine with central roles in immune and inflammatory reactions [22]. Such cytokine has been recently associated with Th1 and Th17 immunity cascades and its increase, along with IFN*γ* and IL-17, demonstrated during *E. granulosus* infection [23]. In a large number of reports [23–26], IL-6 has been found increased during echinococcosis mostly together with Th1 cytokines and aspecific proinflammatory mediators (e.g., CRP). On the other side, the increase of IL-6 has also been reported as an important tool of the Th2 polarization of the host immune defense, thus associated with protection of parasite and to a chronic evolution of the disease [8, 23, 27]. A novel finding of the present study is the increase of IL-6 in the absence of Th1 cytokines and aspecific proinflammatory mediators (e.g., CRP). Interestingly, a major antigen, AgB, of *E. granulosus* has been found to reduce IL-12, but to stimulate IL-6 in an *in vitro* human model [28].Therefore, our isolated increase of IL-6 may be explained with a selective stimulation of such Th2 cytokine associated to a downregulation of Th1 cytokine and other aspecific inflammatory mediators (e.g., CRP).

The significant increase of IL-10 in *Echinococcus*-positive patients evaluated in our study, however, is extremely interesting. This cytokine is universally associated with mechanisms of inhibition and of negative regulation of both T and B cell cascades. It was also observed that IL-10 can promote the survival of the hydatide in patients of echinococcosis [5].

Infection with *Echinococcus* and its pathogenesis have been reported that can represent an excellent model for deepening relations between cellular and humoral mechanisms with immunosuppressive activity [29]. The absence of studies on the activity of B cells regulating the course of echinococcosis is of particular relevance. This cell line regulates all immune responses, and these cells are very often associated with the production of the immunosuppressive cytokine IL-10 [5].

With regard to our observation on the increase of endothelial growth factor (VEGF), there is a lack of data in the literature that may associate the infection by *Echinococcus* in humans with a modification of serum VEGF. However, the increase of this factor could also play an important role in the pathophysiology of the growth of the cysts in various organs. The neoangiogenic trend would certainly encourage the colonization and the growth of the cysts in deep organs. Such a role for VEGF has already been demonstrated for other helminths, such as *Schistosoma* spp. [30].

The significant reduction of the MCP-1 observed in our study could serve as a “marker” of depression of host chemokine, particularly macrophage lineage-driven humoral factor. There are no data in the literature regarding serum MCP-1 level in humans with *Echinococcus* infections. In the BALB/c mice, the experimental infection with *Echinococcus* produced an early serum increase of such chemokine followed by a reduction of serum levels [31].

A decrease of MCP-1 should be associated to an impairment of the role of professional innate defense cells against the treat of *Echinococcus*.

Modifications of the levels of nitrates and nitrites, evaluated as metabolites of nitric oxide, seem to indicate elevated concentrations of these parameters prior to the surgical and medical treatment (T-0) of cysts. The levels of the same analyte were significantly reduced one week (T-1) after the treatment (*p* < 0.05 versus T-0) and again climbed in a substantial way following 1-2 months (T-2) of therapy. This behavior seems to be associated with the high level of nitric oxide to the infection with *Echinococcus*; in fact, its eradication was associated with a drop of the levels of nitric oxide. Other investigators found such postsurgery nitric oxide reduction, which supports the possible involvement of NO in anti-*Echinococcus* activity [32]. Finally, the rise in levels of the analyte after 1-2 months seems to indicate, at least for some patients, either the metastatic spread to other tissues and organs of metacestode, or the regrowth of the larva in the same site.

Paradoxically, nitric oxide, which has been reported to be a parasiticidal molecule, with a direct effect on protoscolices and cyst envelopes [33], should be of benefit to the host in the resistance against the parasite. However, nitric oxide has been found, once released by macrophages, to have a potentially detrimental effect on the lymphocyte function, leading to protein oxidation, lipid peroxidation, and DNA base modifications and strand breaks [34]. A trade-off should be achieved by the parasite, in order to keep the nitrogen radical in check, without inhibiting its release from many host immune cells (e.g., macrophages). Peroxiredoxins are phylogenetically conserved enzymes released by *Echinococcus* and reportedly able to prevent damages to the parasite due to such highly reactive radicals [29].

## 4. Conclusions

The present study sought to spread light on a public health problem in Calabria region. Up to our knowledge, studies on *Echinococcus* infection diffusion in Calabria area are very scarce. Present data clearly demonstrated an endemic spreading in the province of Catanzaro and neighboring territories for such parasitic disease, as well as the novel issue of the number of *Echinococcus*-infected females overcoming the infected males.

On the other side, the possibility of the lack of antibodies in serum samples of some *Echinococcus* patients should call for the identification of novel laboratory markers for the diagnosis of such parasite disease. Therefore, mediators studied in the present work, particularly our novel findings of the increased VEGF and the reduced serum MCP-1 in the studied cases, deserve a further assessment besides the already known increase of IL-10. Overall, the absence of TNF*α* and IFN*γ*, together with the increase of IL-10, may suggest a systemic fair immunodepression caused by the parasite and/or its products. Such subversion of the immune defenses may favor the maintenance of a chronic infection of *Echinococcus* paradoxically inside those organs (e.g., liver and spleen) quite enriched with innate defense cells like macrophages.

## Figures and Tables

**Table 1:** Demoscopic data and laboratory findings of 19 patients from the province of Catanzaro and some neighboring areas studied during the period 2012–2016.

Sub.	Age/sex	Occupational risk	Residency	IDA test (titer)	ELISA	C-reactive protein (ng/ml)	Microscopy for protoscolice hooks	*Echinococcus* real-time PCR and genotype	Therapy
(1) S.A.	46/F	Housewife	Catanzaro	1 : 80	N.d.	<3.03	Pos	Pos G2/G3	Surgery
(2) M.M.	40/F	Housewife	Benestare	Neg	Neg	<3.03	Pos	Pos G2/G3	Surgery
(3) S.E.	29/F	Housewife/farmer	Catanzaro	1 : 80	Pos	<3.03	Pos	Pos G2/G3	Surgery
(4) F.D.	79/M	N.d.	Satriano	1 : 640 Pos	Pos	<3.03	N.d.	N.d.	Albendazole
(5) C.A.	54/F	Seamstress	Cirò	1 : 80	Pos	<3.03	Pos	Pos G2/G3	Surgery
(6) R.C.	67/M	Countryside worker	Crotone	1 : 5120 Pos	Pos	12.50	Pos	Pos G2/G3	Surgery
(7) M.G.	55/F	N.d.	N.d.	1 : 640 Pos	N.d.	<3.03	N.d.	N.d.	N.d.
(8) B.L.	50/F	Countryside worker	Santa Caterina dello Ionio	1 : 2048 Pos	Pos	13.0	Pos	N.d.	Surgery
(9) D.K.	27/F	Student	Chiaravalle	1 : 4096 Pos	Pos	<3.03	N.d.	N.d.	Surgery
(10) M.D.	48/F	Countryside worker	Chiaravalle	1 : 52,428 Pos	Pos	<3.03	N.d.	N.d.	Surgery
(11) M.T.	64/M	N.d.	Caulonia	1 : 320 Pos	N.d.	<3.03	N.d.	N.d.	N.d.
(12) A.E.	76/F	Retired	Cirò	1 : 10,240 Pos	Pos	<3.03	N.d.	N.d.	N.a.
(13) C.D.	61/M	Retired	N.d.	1 : 2048 Pos	Pos	<3.03	N.d.	N.d.	N.a.
(14) F.G.	40/M	N.d.	Mesoraca	1 : 320 Pos	N.d.	14.50	N.d.	N.d.	N.a.
(15) L.M.	57/M	N.d.	Mileto	1 : 10,240 Pos	N.d.	<3.03	N.d.	N.d.	N.a.
(16) D.T.	70/F	N.d.	Strongoli	1 : 160 Pos	Grey zone	12.50	N.d.	N.d.	N.a.
(17) D.F.	86/M	N.d.	Gimigliano	1 : 320 Pos	N.d.	18.00	N.d.	N.d.	N.a.
(18) G.F.	60/M	Driver/farmer	Strongoli	1 : 160 Pos	Pos	17.40	N.d.	N.d.	N.a.
(19) C.L.	48/M	N.d.	San Mauro Marchesato	1 : 160 Pos	N.d.	<3.03	N.d.	N.d.	N.a.

Pos: positive; Neg: negative; N.d.: not done; N.a.: not available.
